# Quercetin and nanoquercetin mitigate high fat diet–induced obesity via lipid modulation, genomic DNA integrity restoration, adipokine regulation, and hepato-pancreatic tissue preservation

**DOI:** 10.1038/s41598-026-41808-5

**Published:** 2026-03-22

**Authors:** Marwa A. Lotify, Sherein S. Abdelgayed, Hanan R.H. Mohamed

**Affiliations:** 1 Zoology Department, Faculty of Science, Cairo University, Giza, Egypt; 2 Pathobiology department, College of Veterinary Medicine, Tuskegee University, Tuskegee, AL 36088 USA; 3 Department of Pathology, Faculty of Veterinary Medicine, Cairo University, Giza, 12211 Egypt

**Keywords:** Flavonoids, Nanoparticles, High-Fat Diet, Lipid Metabolism, Hepatic and Pancreatic Function, Oxidative DNA Damage, Biochemistry, Biotechnology, Drug discovery

## Abstract

Obesity is a global health challenge characterized by excessive fat accumulation and associated with life-threatening comorbidities such as type 2 diabetes, cardiovascular diseases, and certain cancers. Conventional treatments, including lifestyle modification and pharmacotherapy, often have limited long-term efficacy and potential side effects, highlighting the need for safer alternatives. Natural bioactive compounds, such as quercetin, a dietary flavonoid with antioxidant, anti-inflammatory, and metabolic regulatory properties, have emerged as promising anti-obesity agents. However, poor bioavailability limits its therapeutic application, prompting the development of nanoformulations. This study therefore estimated the anti-obesity potential of quercetin and nanoquercetin in a high-fat diet (HFD)-induced obesity model in male Wistar rats. Following acute toxicity testing, 36 rats were divided into six groups: non-obese control, obese HFD control, and non-obese or obese rats orally received quercetin or nanoquercetin at 10% of the safe dose daily for four weeks. Outcomes assessed included body weight, lipid profile, serum total protein, genomic DNA integrity, *Adiponectin* and *Leptin* gene expression, and histological changes in liver and pancreatic tissues. In non-obese rats, quercetin and nanoquercetin did not affect body weight and genomic DNA integrity but improved lipid profiles. Nanoquercetin additionally increased total protein levels. Both compounds upregulated *Adiponectin* expression in the liver, with nanoquercetin also enhancing pancreatic *Adiponectin* expression. Histology revealed preserved tissue architecture. In obese rats, administration of quercetin or nanoquercetin significantly reduced body weight, improved lipid and protein parameters, restored genomic DNA integrity, upregulated *Adiponectin*, downregulated *Leptin*, and markedly improved hepatic and pancreatic histological architecture. Nanoquercetin consistently produced more pronounced effects than quercetin.nIn. These findings demonstrate the therapeutic potential of quercetin, particularly its nanoform, as a multi-targeted anti-obesity agent. Its effects on metabolic regulation, genomic protection, and tissue preservation support further preclinical and clinical studies to explore its role as a safe and effective strategy for managing obesity.

## Introduction

Obesity is a chronic, multifactorial metabolic disorder characterized by excessive fat accumulation, posing serious health risks and reducing quality of life. Its global prevalence has risen sharply among both adults and children, prompting the World Health Organization to recognize it as a major public health concern^1–3^. Obesity is strongly associated with an increased risk of type 2 diabetes, cardiovascular disease, dyslipidemia, nonalcoholic fatty liver disease, and certain cancers, largely driven by chronic inflammation, hormonal imbalances, and metabolic dysfunction^4–6^. It also contributes to adverse mental health issues, including anxiety and depression, amplifying its societal and economic burden^7–9^. Epidemiologically, obesity accounts for approximately 44% of type 2 diabetes cases, 23% of ischemic heart disease cases, and up to 41% of some cancers, and it ranks as the fifth leading risk factor for global mortality, causing at least 2.8 million deaths annually^8,10,11^. These statistics highlight the pressing need for effective, safe, and sustainable interventions to prevent and manage obesity.

Natural bioactive compounds have gained increasing attention as promising candidates for obesity management owing to their favorable safety profiles and broad biological activities. Plant-derived polyphenols, in particular, exhibit antioxidant, anti-inflammatory, and metabolic regulatory properties that may alleviate obesity-related complications while avoiding several limitations associated with conventional anti-obesity pharmacotherapy, such as gastrointestinal side effects, cardiovascular complications, and mood alterations^12,13^. Quercetin, a widely distributed dietary flavonoid present in fruits, vegetables, tea, and red wine, has been shown to improve lipid metabolism, reduce oxidative stress, and modulate adipocyte function^14,15^. Its broad spectrum of biological activities and favorable safety profile support its potential use as an adjunctive or preventive agent in obesity management. Structurally, quercetin is a flavonol aglycone characterized by a diphenylpropane (C6–C3–C6) backbone and multiple hydroxyl groups, which contribute to its potent free radical scavenging capacity^16^.

Despite its therapeutic potential, the clinical application of quercetin is limited by poor water solubility, low oral bioavailability, and extensive metabolism in the gut and liver^17,18^. Nanoformulations, such as nanoquercetin, have been developed to overcome these limitations, offering enhanced solubility, stability, and cellular uptake, which can increase systemic exposure and biological activity^19^. While quercetin has demonstrated efficacy in modulating weight, lipid profiles, and insulin sensitivity^20,21^, studies investigating the anti-obesity potential of nanoquercetin remain scarce, particularly regarding its effects on genomic stability, adipokine gene expression, and histopathological alterations in metabolically active tissues.

In this context, the present study was undertaken to estimate and compare the anti-obesity effects of quercetin and nanoquercetin in a high-fat diet–induced obese Wistar rat model. Using an integrated approach combining biochemical, molecular, and histological analyses, and this study provides novel insights into the potential advantages of nanoquercetin over conventional quercetin, including its impact on lipid homeostasis, adipokine regulation, genomic DNA integrity, and liver and pancreatic tissue preservation.

## Materials and methods

### Chemicals and reagents

Quercetin (≥ 95% purity) was obtained from Sigma-Aldrich (St. Louis, MO, USA). Nanoquercetin was purchased as a ready-to-use formulation from Naqaa Nanotechnology Company (Cairo, Egypt). Its physicochemical properties were previously characterized by El-Sayed et al.^22^, confirming a particle size range of 182–240 nm with uniform morphology and size distribution, ensuring suitability, stability, and consistency for biomedical applications. All other chemicals and reagents used in the experimental procedures were of analytical grade and obtained from standard commercial sources. Colorimetric assay kits for the determination of lipid profile parameters and total protein were sourced from Biodiagnostic Company (Cairo, Egypt) and used according to the manufacturer’s protocols.

### Animal housing and husbandry

Male Wistar rats (Rattus norvegicus), aged 8–10 weeks and weighing 150–180 gram, were obtained from the Animal House of the National Research Centre (Dokki Giza, Egypt). All experimental animals were housed under standardized laboratory conditions in the animal facility of Zoology Department Faculty of Science Cairo University. Rats were maintained in polypropylene cages (dimensions: approximately 40 × 25 × 20 cm), with a maximum of three animals per cage to avoid overcrowding. Cages were bedded with clean, autoclaved wood shavings, which were replaced twice weekly. Animals were kept under controlled environmental conditions, including a temperature of 22 ± 2 °C, relative humidity of 50–60%, and a 12 h light/12 h dark cycle. Rats had free access to standard laboratory chow or high-fat diet, as appropriate, and tap water ad libitum. All husbandry procedures were conducted in accordance with institutional animal care guidelines and the ARRIVE recommendations.

### Animals housing and ethical approval

All procedures involving animals were conducted in accordance with ethical standards for the care and use of laboratory animals following the guidelines of the National Institutes of Health (NIH, 2011) and were approved by the Institutional Animal Care and Use Committee (IACUC) in Egypt, under accreditation number CU/I/F/13/21.

### Randomization and blinding

Animals were randomly assigned to the experimental groups prior to the initiation of treatment to minimize selection bias. Randomization was performed using a simple random allocation method. All experimental procedures were conducted according to the predefined group assignments. Outcome assessments were performed under blinded conditions; specifically, histopathological evaluations and comet assay analyses were carried out by independent investigators who were unaware of the treatment groups. Blinding was maintained throughout data acquisition and analysis to ensure objectivity and to reduce observer bias. All experiments of this study were conducted in accordance with ARRIVE guidelines for preclinical studies to improve transparency and reproducibility of methods and results.

### Determination of safe dose using acute toxicity assay

Acute oral toxicity testing was conducted to determine the safe dose of quercetin and nanoquercetin, following the guidelines outlined in OECD Test Guideline 423 (OECD, 2001). Fifteen male Wistar rats were randomly divided into three groups (*n* = 5 per group): a negative control group, a quercetin-treated group, and a nanoquercetin-treated group. The control group received deionized water orally, while the treated groups were orally administered a single oral dose of quercetin or nanoquercetin at 2000 mg/kg body weight. All animals were observed daily for 14 days for signs of toxicity, behavioral changes, and mortality. Based on the outcomes, one-tenth of the median lethal dose (LD50) was calculated and considered a safe dose for subsequent in vivo experimentation.

### Induction of high fat diet-induced obesity

Obesity was induced using a high-fat diet (HFD) model adapted from Reed et al.^23^, which remains a widely accepted and reproducible method for inducing diet-related metabolic disturbances in rodents. Although originally described over two decades ago, this protocol has been extensively validated and continues to be employed in contemporary obesity research due to its reliability in producing sustained weight gain, dyslipidemia, and metabolic dysfunction. To ensure relevance to current standards, the diet composition and induction duration were selected in line with recent studies using comparable fat content and feeding periods to model human diet-induced obesity^24^. Rats assigned to the obese groups were fed the HFD for the specified induction period prior to initiation of treatment.

The HFD was formulated to provide an energy density of 5.3 kcal/g and consisted of 40% of total caloric content derived from fat, 43% from carbohydrates, and 17% from protein. Lard was used as the sole source of dietary fat, while carbohydrates and proteins were supplied from standard dietary components, including corn starch and casein, respectively. Rats assigned to the obesity model were fed the HFD ad libitum for a continuous period of four weeks. In contrast, the non-obese control group received standard laboratory chow with an energy density of approximately 3.0–3.2 kcal/g. Throughout the dietary induction period, animals were monitored for general health and well-being. Food intake was indirectly assessed through longitudinal body weight measurements, which provide a cumulative indicator of caloric consumption and energy balance. At the end of the fourth week, body weight changes were recorded for all animals to confirm successful obesity induction. Rats exhibiting significant weight gain compared to non-obese controls were classified as obese and subsequently enrolled in the treatment phase of the study.

### Criteria for obesity classification

Obesity was defined using objective and quantifiable criteria rather than subjective assessment. Rats were classified as obese when they exhibited a ≥ 20% increase in body weight compared to age-matched non-obese control rats fed a standard diet, consistent with established definitions for rodent models of diet-induced obesity. This classification was further supported by the presence of dyslipidemia, characterized by significant elevations in serum total cholesterol, triglycerides, and low-density lipoprotein levels, as reported in recent high-fat diet studies. Only animals meeting these predefined criteria were enrolled into the treatment phase of the experiment, ensuring consistency, reproducibility, and scientific rigor.

### Experimental design

Following the successful induction of obesity, a total of thirty-six male Wistar rats, comprising eighteen non-obese and eighteen obese rats, were randomly allocated into six experimental groups (*n* = 6 per group) as follows:

Group 1 (Non-Obese Control): Rats were fed a commercially available standard laboratory chow diet, obtained from El-Nasr Pharmaceutical Chemicals Co., Giza, Egypt. The diet provided an average energy density of approximately 3.0–3.2 kcal/g and consisted of approximately 20–22% protein, 55–60% carbohydrates, and 4–5% fat, along with standard vitamins and mineral supplementation, in accordance with laboratory animal nutritional requirements. Animals had *ad libitum* access to the diet throughout the experimental period.

Group 2 (Non-Obese + Quercetin): Non-obese rats received quercetin orally at a dose equivalent to 10% of the safe dose determined by acute oral toxicity testing, alongside the standard diet, for a period of 4 weeks.

Group 3 (Non-Obese + Nanoquercetin): Non-obese rats received nanoquercetin at the same dose level (10% of the determined safe dose) via oral gavage daily for 4 weeks while continuing on the standard diet.

Group 4 (Obese Control): Obese rats continued to receive the HFD and were administered deionized water orally for 4 weeks to serve as the untreated obese control.

Group 5 (Obese + Quercetin): Obese rats were treated with quercetin orally at a dose equivalent to 10% of the LD50 determined through the acute oral toxicity assay. Treatment continued daily for 4 weeks while the rats remained on the HFD.

Group 6 (Obese + Nanoquercetin): Obese rats received nanoquercetin orally at 10% of the LD50 dose, as established by acute toxicity testing. The treatment lasted 4 weeks in parallel with continued HFD feeding.

All treatments (quercetin, nanoquercetin, or vehicle) were administered via oral gavage once daily using a gastric feeding needle to ensure accurate dosing. During the treatment period, rats were monitored for food intake, physical appearance, behavior, and body weight. At the end of the 4-week treatment period, all animals were fasted overnight, euthanized under anesthesia, and samples of blood, liver, and pancreas were collected for further biochemical, molecular, and histological analyses.

### Body weight and sample collection

Body weight of all rats was monitored and recorded three times per week throughout the experimental period to evaluate changes related to dietary and treatment interventions. At the end of the 4-week treatment phase, animals were fasted overnight to stabilize metabolic parameters. On the following day, rats were anesthetized using inhaled isoflurane (1–4%) in an induction chamber, followed by maintenance at 2% via a nose cone to ensure a surgical plane of anesthesia. Once deep anesthesia was confirmed by the absence of pedal and corneal reflexes, euthanasia was performed by exsanguination via cardiac puncture, in accordance with the ethical guidelines approved by the Institutional Animal Care and Use Committee (CU-IACUC).

Blood samples were collected immediately through cardiac puncture using sterile syringes. The collected blood was transferred into clean, dry tubes and allowed to clot at room temperature, then centrifuged at 3,000 rpm for 10 min to separate the serum. The obtained serum was stored at−20 °C until used for biochemical analysis of lipid profile and total protein using standard colorimetric assay kits. Liver and pancreas tissues were carefully excised, rinsed with cold phosphate-buffered saline (PBS, pH 7.4) to remove residual blood, and gently blotted dry. Each organ was divided into two portions: one was immediately fixed in 10% neutral-buffered formalin for histopathological examination, while the other was snap-frozen in liquid nitrogen and stored at−80 °C for subsequent molecular analyses, including gene expression and DNA damage assessment.

### Biochemical analyses of serum lipid profile and total protein

At the end of the experimental period, serum samples were analyzed to assess the effect of quercetin and nanoquercetin administration on lipid metabolism and protein synthesis in both non-obese and obese rats (Krauss et al., 2000). The serum concentrations of lipid profile including total cholesterol, low-density lipoprotein, high-density lipoprotein and triglycerides were measured in (mg/dL), while total protein was measured in g/dL using commercially available colorimetric diagnostic kits (Biodiagnostic, Cairo, Egypt). The assays were conducted in accordance with the manufacturer’s instructions. For each test, serum samples were thawed at room temperature, and approximately 200 µL of serum was used per assay. Colorimetric reactions were carried out using specific enzymatic reagents provided in the kits, and absorbance was measured using a UV-Visible spectrophotometer at wavelengths specified by the manufacturer (e.g., 500–546 nm for lipid profile parameters and 540 nm for total protein).

### Estimation of genomic DNA integrity using the alkaline comet assay

The effect of quercetin and nanoquercetin on genomic DNA integrity in hepatic and pancreatic tissues of both obese and non-obese rats was evaluated using the alkaline single-cell gel electrophoresis (Comet) assay, a highly sensitive technique for detecting DNA strand breaks at the individual cell level^25,26^. A small portion of liver and pancreas tissues was gently homogenized to obtain a clear single-cell suspension. From this suspension, 10 µL of cell suspension was mixed with 5% low-melting-point agarose, and the mixture was carefully layered onto pre-coated microscope slides containing a 1% normal-melting-point agarose base layer. The slides were allowed to solidify at 4 °C for a few minutes. Once gelled, the slides were immersed in cold lysis solution (2.5 M NaCl, 100 mM EDTA, 10 mM Tris-HCl, pH 10, freshly supplemented with 1% Triton X−100 and 10% DMSO) and incubated at 4 °C for 24 h. This step lysed the cells and removed proteins and membranes, leaving behind nucleoids composed of supercoiled DNA. Following lysis, the slides were transferred to an alkaline electrophoresis buffer (300 mM NaOH, 1 mM EDTA, pH > 13) and incubated for 15 min to allow DNA unwinding and the expression of alkali-labile sites as strand breaks. Electrophoresis was conducted at 25 V and 300 mA for 30 min under constant cooling. After electrophoresis, the slides were gently neutralized in 0.4 M Tris-HCl buffer (pH 7.5) and rinsed with distilled water. DNA was then stained with ethidium bromide (20 µg/mL), and slides were visualized under a fluorescence microscope equipped with a suitable filter set (excitation: 510 nm, emission: 590 nm). For each sample, 50 randomly selected nuclei were examined using Comet Score™ software (TriTek Corp., USA). Three primary comet parameters were quantified as indicators of DNA damage: Tail length (µm), %DNA in tail and tail moment. These parameters were analyzed to determine the extent of genomic instability and to evaluate the genoprotective or genotoxic effects of quercetin and nanoquercetin on liver and pancreatic tissues.

### Analysis of *Adiponectin* and *Leptin* gene expression

Total RNA was extracted from approximately 50–100 mg of Hepatic and pancreatic tissue using TRIzol^®^ Reagent (Invitrogen, Carlsbad, CA, USA). According to the manufacturer’s protocol, tissues were homogenized in TRIzol, followed by phase separation using chloroform, and RNA was precipitated with isopropanol. The RNA pellet was washed with 75% ethanol, air-dried, and re-dissolved in RNase-free water. The purity and concentration of the extracted RNA were assessed spectrophotometrically using a NanoDrop spectrophotometer (Thermo Scientific, USA). The A260/A280 ratio was used to evaluate RNA purity, and samples with ratios between 1.8 and 2.0 were considered acceptable for downstream applications. For complementary DNA (cDNA) synthesis, 1 µg of total RNA was reverse-transcribed using a RevertAid First Strand cDNA Synthesis Kit (Thermo Scientific, USA), according to the manufacturer’s instructions. The reaction mixture included random hexamer primers and was incubated at 42 °C for 60 min, followed by enzyme inactivation at 70 °C for 5 min.

Quantitative real-time PCR (qRT-PCR) was conducted using SYBR™ Green PCR Master Mix (Applied Biosystems, Foster City, CA, USA) in a final volume of 12 µL per reaction. Amplification was performed on a StepOnePlus™ Real-Time PCR System (Applied Biosystems, USA). Gene-specific primers for *Adiponectin*: 5′- gcactggcaagttctactgcaa 3′ 5′-gtaggtgaagagaacggccttgt−3 and *Leptin*: 5′-aggtggaggtgaactggarcggg−3′ 5′-ggcccacaaagtcctctcagcac3′ were used, with sequences adopted from previously validated studies by Joffin et al.^27^. and Joffin et al.^28^. Each reaction was run in triplicate. The *β-actin* gene was used as a housekeeping reference gene to normalize the expression levels of the target genes. The relative expression of each gene was calculated using the 2^−ΔΔCt method^29^, and results were expressed as fold changes relative to the non-obese control group.

### Histopathological examination of hepatic and pancreatic tissues

Samples of liver and pancreatic tissues were immediately fixed in 10% neutral buffered formalin to preserve tissue architecture. After fixation, tissues were processed through a standard series of graded ethanol concentrations for dehydration, cleared in xylene, and then embedded in paraffin wax using an automated tissue processor. Paraffin-embedded tissues were then sectioned at a thickness of 5 μm using a rotary microtome. The sections were mounted on glass slides, dried, and subsequently stained with hematoxylin and eosin following the protocol described by Jensenv^30^,. The stained sections were examined under a light microscope (Leica microscope) to evaluate histopathological alterations in the liver and pancreas tissues. Representative images were captured using a digital imaging system for documentation and comparative analysis across groups.

### Statistical analysis

Statistical analysis was performed using one-way analysis of variance (ANOVA) to compare differences among experimental groups, as the study design involved a single independent factor with multiple levels. Prior to ANOVA, the normality of data distribution was assessed using the Shapiro–Wilk test, and homogeneity of variances was evaluated using Levene’s test; all datasets met the assumptions required for parametric analysis. Post hoc comparisons were carried out using Duncan’s multiple range test to determine differences between group means. This test was chosen for its applicability in exploratory experimental studies aimed at identifying treatment-related biological effects. A p-value < 0.05 was considered statistically significant. Statistical analyses were restricted to predefined outcome measures to minimize multiplicity, and results were interpreted with emphasis on biological relevance rather than strict inferential generalization.

## Results

### Safe dose of quercetin and nanoquercetin

Acute oral toxicity testing was performed according to OECD Guideline 423. As displayed in Table 1 single oral dose of quercetin or nanoquercetin at 2000 mg/kg body weight did not produce any mortality, behavioral abnormalities, or signs of toxicity in the treated rats during the 14-day observation period. All animals appeared healthy, remained active, and showed normal grooming, feeding, and locomotor behavior throughout the study. Although no quantitative measurements were recorded, these qualitative observations indicate the absence of any overt toxicity. Based on these findings, the median lethal dose (LD50) of both quercetin and nanoquercetin was estimated to be greater than 2000 mg/kg, supporting their low acute toxicity. Consequently, 10% of the LD50 (200 mg/kg body weight) was selected as a safe and effective dose for subsequent experimental treatments.

**Table 1:** Acute oral toxicity assessment of quercetin and nanoquercetin according to OECD Test Guideline 423.

Treatment	Dose (mg/kg bw)	Number of rats	Mortality (0–14 days)	Clinical signs observed	Onset and duration of signs	Body weight change	Necropsy findings	GHS toxicity category
Negative control	Deionized water	5	0/5	None observed	Not applicable	Normal weight gain	No gross abnormalities	Category 5/Unclassified
Quercetin	2000 mg/kg	5	0/5	Mild lethargy (if any)	Transient (first 24 h)	Normal weight gain	No gross abnormalities	Category 5/Unclassified
Nanoquercetin	2000 mg/kg	5	0/5	None observed	Not applicable	Normal weight gain	No gross abnormalities	Category 5/Unclassified

- Acute oral toxicity was evaluated according to OECD Test Guideline 423 (Acute Toxic Class Method).

- Animals were observed continuously during the first 4 h post-administration and daily thereafter for 14 days.

- Clinical signs, mortality, body weight changes, and gross pathological findings were recorded.

- No treatment-related mortality was observed up to the highest tested dose.

- Based on these findings, quercetin and nanoquercetin were classified under GHS Category 5/unclassified for acute oral toxicity.

### Effect of quercetin and nanoquercetin on body weight

**Fig. 1:**
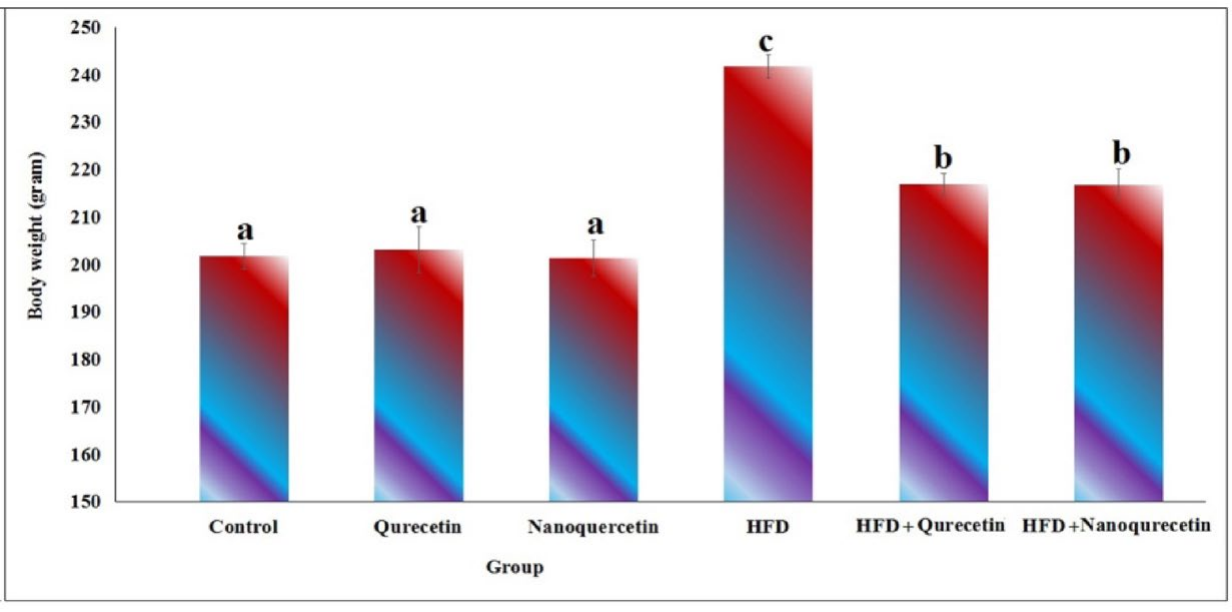
Therapeutic influence of Quercetin and Nanoquercetin on body weight in obese and non-obese rats. Six rats were used per each group and data are presented as mean ± SD. Statistical analysis was performed using one-way ANOVA followed by Duncan’s post hoc test. Different letters indicate statistically significant differences between untreated and treated groups at *p* < 0.001.

As illustrated in Fig. 1, no statistically significant differences in body weight were observed in non-obese rats administered quercetin or nanoquercetin at a dose level of 200 mg/kg b.w. (equivalent to 10% of the LD50 determined from the acute toxicity test) compared with the non-obese control group, indicating the absence of detectable effects on body weight under non-obese conditions. In contrast, rats fed a HFD for four weeks exhibited a significant increase in body weight compared with the non-obese control group (*p* < 0.001), confirming successful induction of obesity (Fig. 1). Oral administration of quercetin or nanoquercetin to HFD-fed rats for four weeks resulted in a significant reduction in body weight compared with untreated obese rats (*p* < 0.001). However, body weight in quercetin- or nanoquercetin-treated obese rats remained significantly higher than that of the non-obese control group (Fig. 1).

### Effect of quercetin and nanoquercetin on serum lipid profile

As shown in Table 2, daily oral administration of quercetin or nanoquercetin (200 mg/kg body weight; 10% of the LD50) for four weeks in non-obese rats resulted in statistically significant changes in serum lipid parameters compared with the untreated non-obese control group, including reductions in total cholesterol, triglycerides, and LDL levels, along with an increase in HDL level (*p* < 0.001). Nanoquercetin produced more pronounced effects on total cholesterol, triglycerides, and HDL levels compared with quercetin.

**Table 2:** Influence of quercetin and nanoquercetin oral administration at a dose level of 200 mg/kg (10% LD50 dose) on serum lipid profile of rats fed with normal- and high fat-diet.

Group	Serum lipid profile			
	Total Cholesterol (mg/dL)	Triglycerides (mg/dL)	HDL (mg/dL)	LDL (mg/dL)
Negative control	96.43 ± 3.48 ^c^	92.70 ± 2.25 ^c^	30.57 ± 1.85 ^b^	50.06 ± 0.82 ^b^
Quercetin	68.24 ± 1.90 ^b^	84.07 ± 5.40 ^b^	30.61 ± 2.39 ^b^	19.07 ± 1.37 ^a^
Nano Quercetin	59.97 ± 6.60 ^a^	54.65 ± 4.24 ^a^	35.26 ± 1.63 ^c^	18.31 ± 4.55 ^a^
High Fat Diet (HFD)	123.28 ± 2.48 ^d^	107.59 ± 5.04 ^d^	24.33 ± 1.36 ^a^	60.72 ± 4.07 ^c^
HFD + Quercetin	96.04 ± 4.13 ^c^	92.53 ± 2.48 ^c^	31.29 ± 1.25 ^b^	50.03 ± 0.73 ^b^
HFD + Nano Quercetin	93.65 ± 2.14 ^c^	95.63 ± 3.08 ^c^	29.06 ± 1.80 ^b^	47.37 ± 2.47 ^b^
ANOVA	F = 139.50 *p* < 0.001	F = 59.27 *p* < 0.001	F = 12.20 *p* < 0.001	F = 123.02 *p* < 0.001

• Results are expressed as mean ± SD.

• Results were analyzed using one-way analysis of variance followed by Duncan’s test to test the similarity between the control and treated groups.

• Means with different letters indicates statistical significant difference at *p* < 0.001 between the compared cells in the same column.

In contrast, rats fed a HFD for four weeks exhibited marked dyslipidemia, characterized by significantly elevated serum total cholesterol, triglycerides, and LDL levels and a reduced HDL level compared with the non-obese control group (*p* < 0.001), confirming the induction of obesity-associated lipid disturbances (Table 2). Treatment of HFD-fed rats with quercetin or nanoquercetin for four weeks significantly improved these lipid abnormalities compared with untreated obese rats (*p* < 0.001), as reflected by decreases in total cholesterol, triglycerides, and LDL levels and an increase in HDL level. However, lipid parameters in treated obese rats remained statistically different from those of the non-obese control group (Table 2).

### Effect of quercetin and nanoquercetin on serum total protein level

**Fig. 2:**
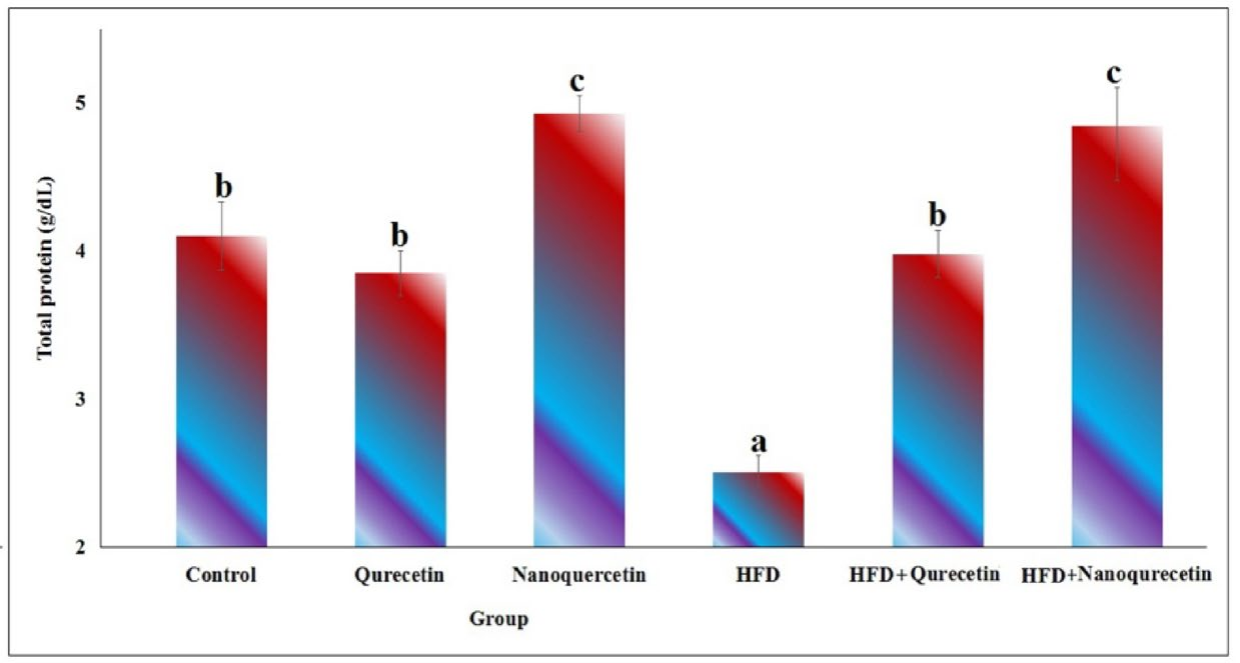
Total protein levels (g/dL) in high fat diet obese and non-obese rats after oral administration of Quercetin and Nanoquercetin at a dose level of 200 mg/kg. Six rats were used per each group and data are expressed as mean ± SD. Statistical analysis was performed using one-way ANOVA followed by Duncan’s post hoc test. Different letters indicate statistically significant differences between control and treated groups at *p* < 0.001.

As displayed in Fig. 2, oral administration of quercetin at a dose level of 200 mg/kg b.w. for four weeks in non-obese rats did not significantly affect serum total protein levels compared with the non-obese control group. In contrast, nanoquercetin administration at the same dose and duration produced a significant increase in total protein levels compared with untreated non-obese rats (*p* < 0.001).

Feeding rats a HFD for four weeks caused a significant reduction in serum total protein levels in untreated obese rats compared with non-obese controls (*p* < 0.001), indicating altered protein metabolism associated with obesity. Treatment of obese rats with quercetin or nanoquercetin significantly improved total protein levels compared with untreated obese rats (*p* < 0.001). Quercetin partially restored total protein toward the levels of the non-obese control group, while nanoquercetin produced a more pronounced increase compared with untreated obese and non-obese rats (Fig. 2), suggesting enhanced protein synthesis or reduced protein degradation in response to nanoquercetin administration.

### Effect of quercetin and nanoquercetin on genomic DNA integrity

**Fig. 3:**
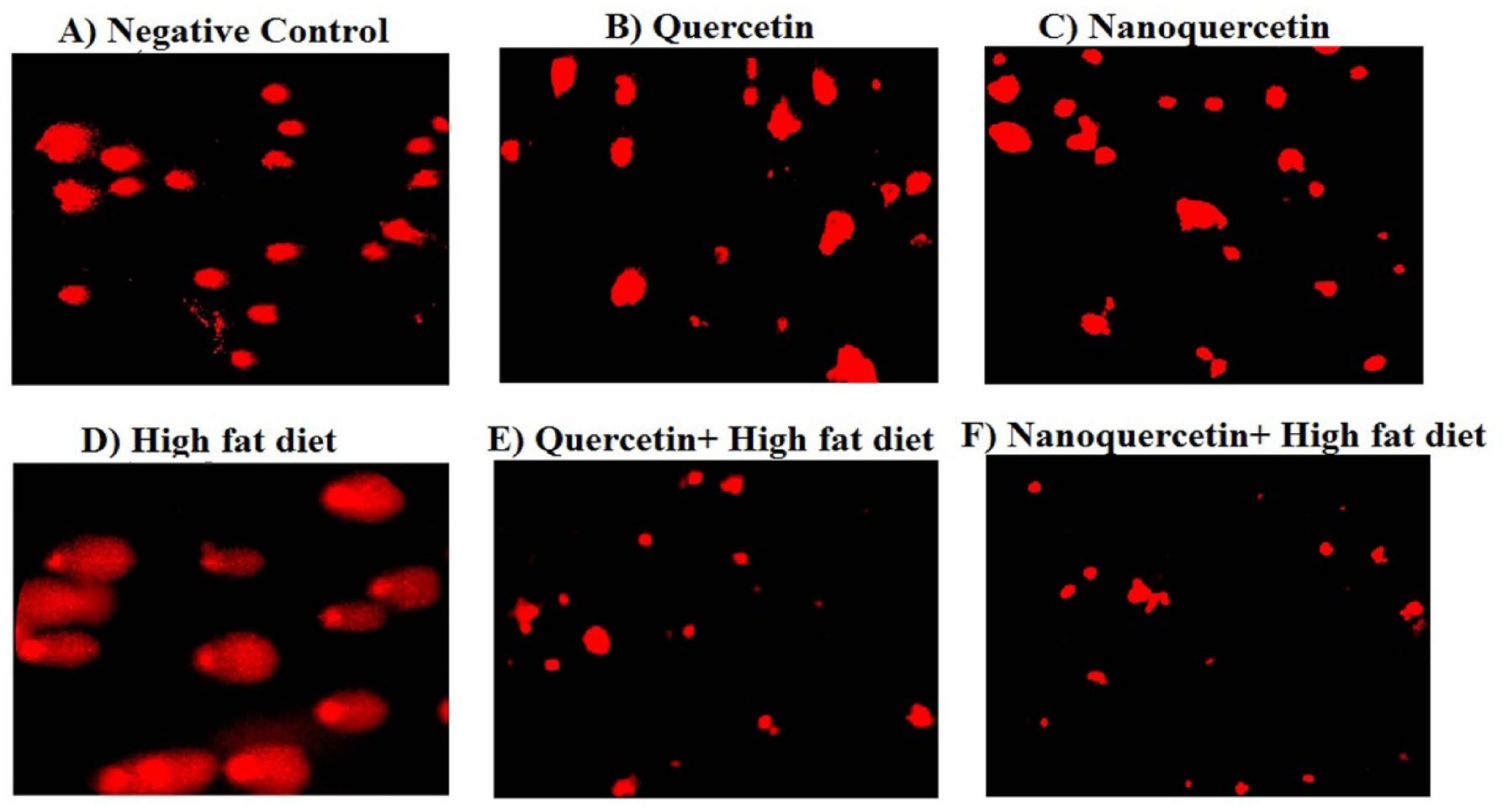
Representative Comet assay images showing nuclei with minimal DNA migration in normal non-obese rats and in obese rats administered Quercetin or Nanoquercetin (200 mg/kg), whereas pronounced DNA fragmentation and comet tail formation are observed in high-fat diet (HFD)–induced obese rats. Images were captured at 200× magnification.

The results of the alkaline comet assay, summarized in Table 3 and illustrated in Fig. 3, showed that oral administration of quercetin or nanoquercetin (200 mg/kg b.w.; 10% of the LD50) for four weeks did not induce DNA damage in the hepatic or pancreatic tissues of non-obese rats. No significant differences were observed in tail length, %DNA in tail, or tail moment compared with the non-obese control group, indicating that both compounds did not adversely affect genomic DNA under non-obese conditions.

**Table 3:** Impact of quercetin and nanoquercetin oral administration at a dose level of 200 mg/kg (10% LD50 dose) on genomic DNA integrity in the liver and pancreas of rats fed with normal- and high fat-diet.

Group	Liver			Pancreas		
	Tail length (px)	%DNA in tail	Tail moment	Tail length (px)	%DNA in tail	Tail moment
Negative control	3.37 ± 0.75 ^a^	19.84 ± 0.96 ^a^	0.79 ± 0.12 ^a^	4.27 ± 0.41 ^a^	13.16 ± 1.06 ^a^	0.67 ± 0.07 ^a^
Quercetin	2.98 ± 0.61^a^	18.01 ± 4.00 ^a^	0.71 ± 0.34 ^a^	4.27 ± 0.29 ^a^	15.37 ± 1.96 ^a^	0.74 ± 0.06 ^a^
Nanoquercetin	3.65 ± 0.74 ^a^	16.17 ± 2.86 ^a^	0.67 ± 0.13 ^a^	4.97 ± 0.50 ^a^	16.04 ± 1.63 ^a^	0.75 ± 0.14 ^a^
High Fat Diet (HFD)	8.88 ± 0.43 ^b^	18.67 ± 4.24 ^a^	1.83 ± 0.08 ^b^	19.83 ± 4.16 ^b^	35.83 ± 3.99 ^b^	6.95 ± 2.36 ^b^
HFD + Quercetin	3.75 ± 0.51^a^	20.63 ± 6.98 ^a^	0.79 ± 0.34 ^a^	3.93 ± 0.93 ^a^	16.33 ± 3.11 ^a^	0.62 ± 0.28 ^a^
HFD + Nanoquercetin	3.65 ± 0.74 ^a^	16.17 ± 2.87 ^a^	0.67 ± 0.13 ^a^	4.97 ± 0.17 ^a^	16.16 ± 2.61 ^a^	0.69 ± 0.08 ^a^
ANOVA	F = 35.59 *p* < 0.001	F = 0.62 *p* > 0.05	F = 12.75 *p* < 0.001	F = 37.90 *p* < 0.001	F = 30.52 *p* < 0.001	F = 20.73 *p* < 0.001

• Results are expressed as mean ± SD.

• Results were analyzed using one-way analysis of variance followed by Duncan’s test to test the similarity between the control and treated groups.

• Means with different letters indicates statistical significant difference at *p* < 0.001 between the compared cells in the same column.

In contrast, rats fed a HFD for daily four weeks exhibited marked genomic instability, as evidenced by a significant increase (*p* < 0.001) in all comet assay parameters in liver and pancreatic tissues compared with non-obese control rats, confirming the genotoxic effects of diet-induced obesity. Treatment of obese rats with quercetin or nanoquercetin at the same dose significantly reduced tail length, %DNA in tail, and tail moment compared with untreated obese rats (*p* < 0.001). These results indicate that both treatments mitigated obesity-associated DNA damage. Representative comet images depicting nuclei with intact DNA and varying degrees of damage are shown in Fig. 3.

### Effect of quercetin and nanoquercetin on *Adiponectin* and *Leptin* gene expression

As displayed in Table 4, oral administration of quercetin or nanoquercetin (200 mg/kg b.w.) for daily four weeks in non-obese rats did not significantly affect *Leptin* gene expression in liver or pancreatic tissues compared with the untreated non-obese control group. However, both treatments resulted in a significant upregulation of *Adiponectin* expression in hepatic tissues (*p* < 0.001). Notably, nanoquercetin also produced a significant increase in adiponectin expression in pancreatic tissues, whereas quercetin did not elicit a similar effect (Table 4). These changes were quantified using the ΔΔCt method with normalization to the selected *GAPDH* housekeeping gene, and all reactions were performed in technical triplicates to ensure data reliability.

**Table 4:** Effect of quercetin and nanoquercetin oral administration at a dose level of 200 mg/kg (10% LD50 dose) on expression level of *Adiponectin* and *Leptin* genes in the liver and pancreas of rats fed with normal- and high fat-diet.

Group	Liver		Pancreas	
	Adiponectin gene expression	Leptin gene expression	Adiponectin gene expression	Leptin gene expression
Negative control	1.00 ± 0.00 ^a^	1.00 ± 0.00 ^a^	1.00 ± 0.00 ^c^	1.00 ± 0.00 ^a^
Quercetin	10.29 ± 0.97 ^e^	0.85 ± 0.04 ^a^	0.97 ± 0.02 ^bc^	1.15 ± 0.04 ^a^
Nano Quercetin	8.47 ± 1.27 ^d^	0.93 ± 0.02 ^a^	1.26 ± 0.14 ^d^	1.09 ± 0.06 ^a^
High Fat Diet (HFD)	2.84 ± 0.44 ^b^	9.54 ± 0.43 ^c^	0.51 ± 0.06 ^a^	6.27 v 0.78 ^c^
HFD + Quercetin	6.45 ± 0.49 ^c^	1.59 ± 0.16 ^b^	0.86 ± 0.05 ^b^	2.72 ± 0.41 ^b^
HFD + Nano Quercetin	10.08 ± 0.46 ^e^	1.14 ± 0.05 ^a^	1.22 ± 0.04 ^d^	1.19 ± 0.03 ^a^
ANOVA	F = 84.37 *p* < 0.001	F = 997.14 *p* < 0.001	F = 49.57 *p* < 0.001	F = 99.26 *p* < 0.001

• Results are expressed as mean ± SD.

• Results were analyzed using one-way analysis of variance followed by Duncan’s test to test the similarity between the control and treated groups.

• Means with different letters indicates statistical significant difference at *p* < 0.001 between the compared cells in the same column.

In contrast, HFD-fed rats exhibited pronounced dysregulation of adipokine gene expression, characterized by a significant decrease in *Adiponectin* and a significant increase in *Leptin* expression in both liver and pancreatic tissues compared with non-obese control rats (*p* < 0.001), consistent with obesity-associated metabolic and inflammatory disturbances (Table 4). Treatment of obese rats with quercetin or nanoquercetin at the same dose for the same duration significantly ameliorated these alterations (*p* < 0.001), reflected by increased adiponectin and decreased *Leptin* expression relative to untreated obese rats. Nanoquercetin produced a more pronounced effect, partially restoring leptin expression toward the non-obese control levels and further enhancing *Adiponectin* expression above baseline non-obese values (*p* < 0.001). While these fold-changes are biologically notable, the results were carefully normalized and technically validated to ensure accuracy, and interpretations are made cautiously to reflect relative changes in adipokine signaling rather than absolute biological thresholds.

### Effect of quercetin and nanoquercetin on architecture of liver and pancreas tissues

**Fig. 4:**
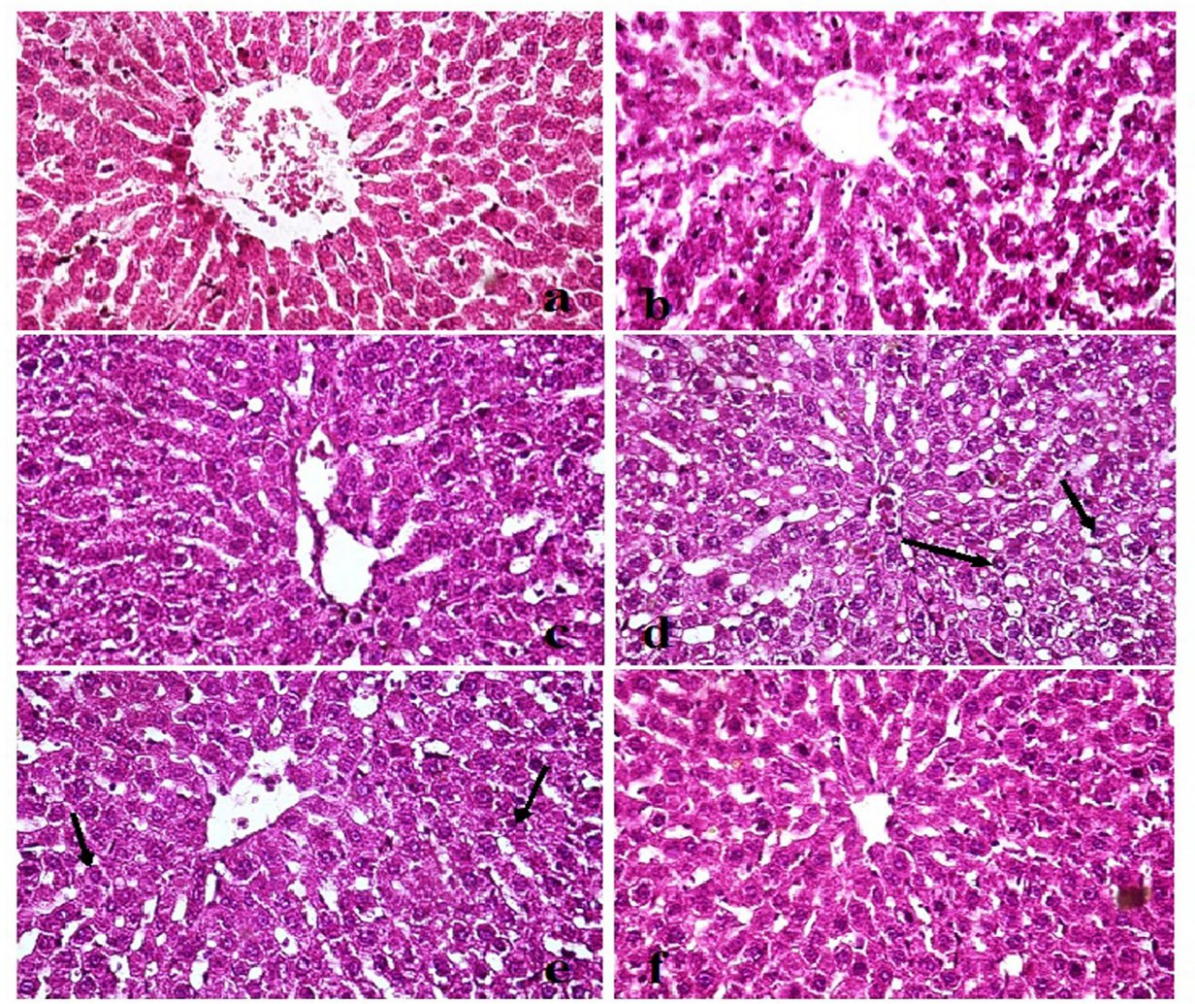
Photomicrographs of liver tissues from different experimental groups stained with H&E X400 showing: (**a**) Control normal group (0), (**b**) Quercitin administered non-obese group with apparently healthy liver parenchyma (0), (**c**) Nano-Quercitin administered non-obese group with no histological lesions (0), (**d**) High fat diet (HFD) obese group with diffuse vacuolar degenerated hepatocytes (arrows) (+++), e) HFD + Quercitin group with focal sporadic vacuolar degeneration (arrows) (+), and f) HFD + Nano-Quercitin group with no hepatic damage (0).

Histological examination of liver sections from the untreated non-obese control group showed well-preserved hepatic architecture, with polygonal hepatocytes arranged in regular cords and intact portal triads (Fig. 4a). Liver tissues from non-obese rats treated with quercetin or nanoquercetin (200 mg/kg b.w.) exhibited similar histological features, with no detectable lesions or structural abnormalities, indicating good tissue tolerance (Fig. 4b and c). In contrast, liver sections from HFD-fed obese rats displayed marked pathological changes, including diffuse vacuolar degeneration of hepatocytes, consistent with hepatic steatosis and lipid accumulation (Fig. 4d). Treatment of obese rats with quercetin resulted in partial improvement, with vacuolar degeneration limited to focal areas (Fig. 4e), whereas nanoquercetin administration produced more extensive preservation of hepatic architecture, with the majority of hepatocytes appearing structurally intact and reduced vacuolar changes (Fig. 4f).

**Fig. 5:**
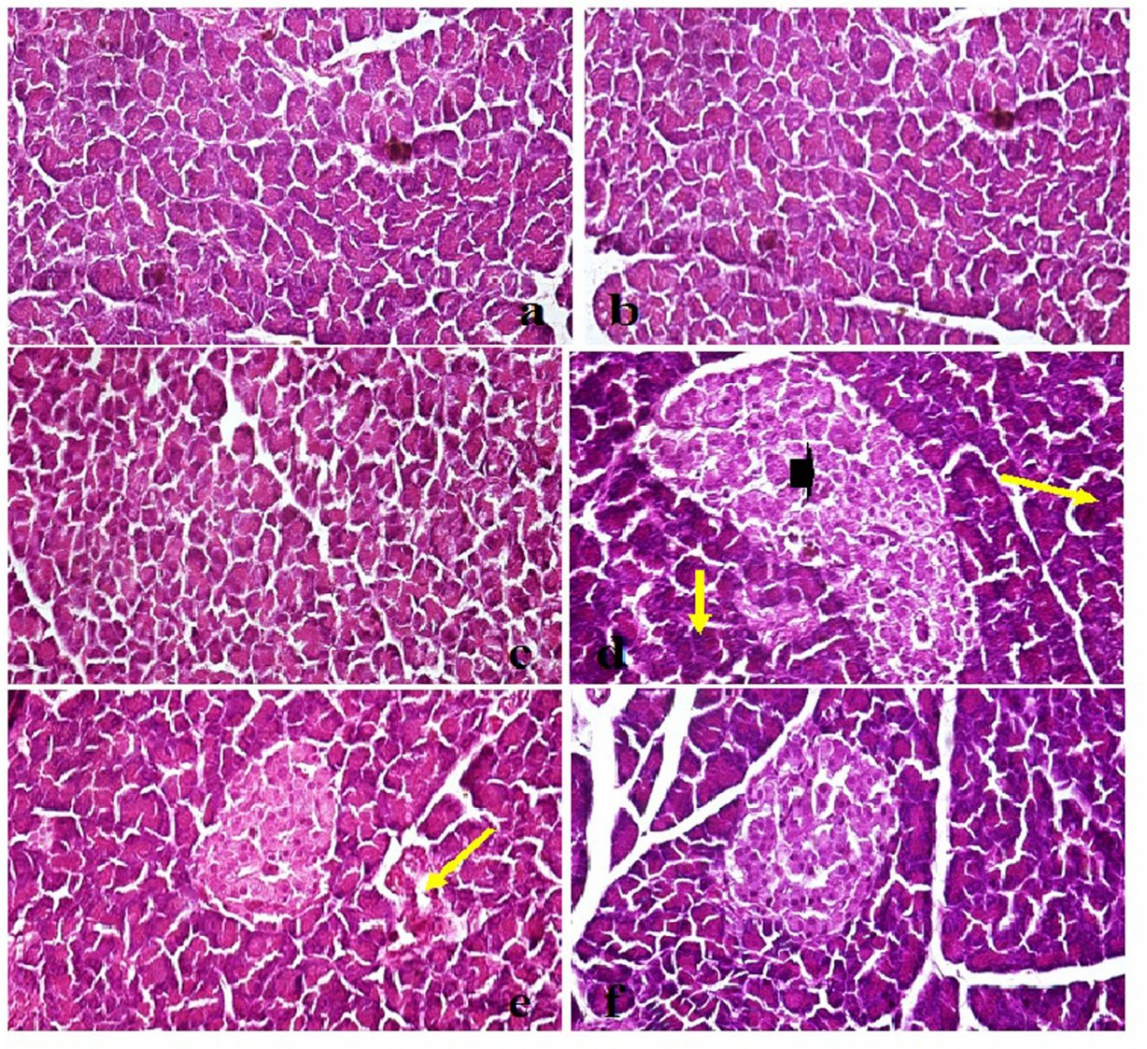
Photomicrographs of Pancreas from different experimental groups stained with H&E X400 showing: (**a**) Control normal group (0), (**b**) Quercitin group with apparently healthy pancreatic parenchyma (0), (**c**) Nano-Quercitin group with no histological lesions (0), (**d**) High fat diet (HFD) obese group with severe hyperplasia in pancreatic islets (arrow head) and necrosed pancreatic acini (arrows) (+++), e) HFD + Quercitin group with normal pancreatic islets and mild damage in the pancreatic acini (arrow) (+), and f) HFD + Nano-Quercitin group with no pancreatic damage (0).

Microscopic evaluation of pancreatic tissue from non-obese control rats revealed tightly packed acini and well-organized islets of Langerhans (Fig. 5a). Pancreatic tissues from non-obese rats administered quercetin or nanoquercetin maintained comparable architecture, with no observable histological alterations (Fig. 5b, c). Conversely, HFD-fed obese rats exhibited notable histopathological changes, including islet hyperplasia and necrosis of pancreatic acinar cells, indicative of diet-induced pancreatic injury (Fig. 5d). Quercetin treatment of obese rats led to moderate improvement, with only mild acinar cell damage observed (Fig. 5e), while nanoquercetin administration was associated with a more marked preservation of pancreatic structure and reduced acinar cell degeneration (Fig. 5f).

## Discussion

Obesity is a complex metabolic disorder characterized by dysregulated lipid metabolism, chronic low-grade inflammation, oxidative stress, and multi-organ dysfunction. Although several pharmacological interventions are currently available for obesity management, their long-term efficacy is often limited by adverse effects, high cost, and poor adherence, underscoring the need for safer adjunct or alternative strategies^31–34^. In this context, plant-derived bioactive compounds have attracted increasing attention due to their pleiotropic metabolic effects and favorable safety profiles^35^.

Quercetin, a widely studied dietary flavonoid, has been reported to modulate lipid metabolism, oxidative stress, and inflammatory signaling pathways relevant to obesity. However, its clinical translation remains limited by low solubility, rapid metabolism and poor bioavailability^36^. To overcome these limitations, nanoparticle-based delivery systems, such as nanoquercetin, have been developed to improve quercetin stability, gastrointestinal absorption, and tissue-targeting capabilities^37,38^. In this context, the current study was conducted to comparatively estimate the metabolic and molecular effects of quercetin and nanoquercetin in a HFD induced obesity model in rats.

**Fig. 6:**
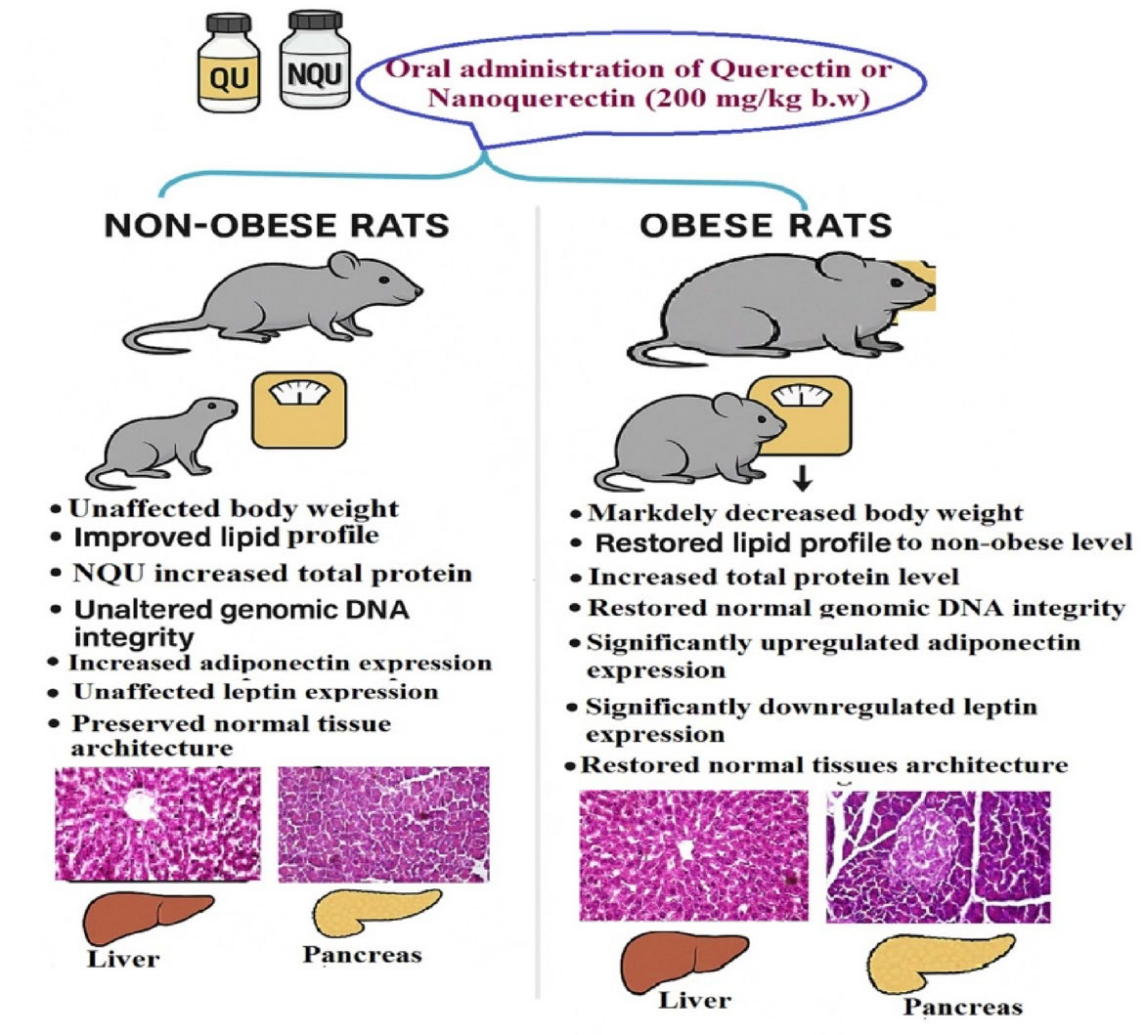
Schematic diagram showing therapeutic modulation of obesity-associated parameters by Quercetin and nanoquercetin in HFD obese and non-obese rats.

The findings of this study provide the first comparative in *vivo* evidence demonstrating that nanoformulation of quercetin enhances its biological activity in a HFD–induced obesity model.When administered at an equivalent dose, nanoquercetin produced more pronounced improvements than native quercetin across several assessed parameters, including lipid profile regulation, modulation of adipokine gene expression, attenuation of DNA damage markers, and preservation of hepatic and pancreatic histological features, as summarized in Fig. 6. These results suggest that nanoformulation may enhance the functional efficacy of quercetin in experimental obesity, although further mechanistic and pharmacokinetic investigations are required to substantiate these observations.

Importantly, the dose used in this study was selected based on acute oral toxicity testing rather than pharmacological dose–response optimization. According to OECD guidelines (TG 423/425), no mortality or observable signs of toxicity were detected up to the limit dose, indicating an adequate safety margin. Therefore, a dose equivalent to 10% of the estimated LD50 (200 mg/kg) was chosen as a safe, sub-toxic dose for repeated administration. This strategy is widely applied in experimental research to ensure animal safety and to avoid systemic toxicity that could interfere with biochemical, molecular, and histopathological assessments^39^. The main objective of the present study was to investigate the biological and mechanistic effects of the tested compound under non-toxic conditions, rather than to determine an optimized pharmacological or translational dose. Pharmacological dose optimization requires detailed pharmacokinetic and efficacy evaluations, which are typically performed at later stages of research and were beyond the scope of this study. Accordingly, dose selection based on acute toxicity data alone is scientifically appropriate, methodologically justified, and consistent with established practices in similar in vivo studies^39,40^.

Our findings of acute toxicity test demonstrated that both quercetin and nanoquercetin were well tolerated in non-obese rats, with no detectable biochemical alterations, histopathological lesions, or genotoxic findings. These results align with previous studies demonstrating the favorable safety profile of quercetin and indicate that nanoformulation did not introduce detectable toxicity at the tested dose^15,41^. Furthermore, repeated oral administration of quercetin or nanoquercetin (200 mg/kg body weight) daily for four weeks confirmed their safety in healthy rats. Serum total protein levels remained within normal ranges following quercetin treatment, whereas nanoquercetin induced a significant increase, indicating a potential enhancement of hepatic synthetic function without toxic effects. Lipid profiles, including total cholesterol, triglycerides, LDL, and HDL, improved significantly in treated rats, reflecting beneficial metabolic modulation. Comet assay analysis revealed no DNA damage in liver or pancreatic tissues, supporting genomic stability and the absence of genotoxic effects. Additionally, gene expression analysis showed no abnormal changes in leptin levels and a favorable upregulation of adiponectin, particularly in the nanoquercetin group. Collectively, our findings demonstrate that quercetin and nanoquercetin are safe for repeated administration in healthy animals and may provide metabolic and molecular benefits without harmful side effects^42,43^.

The anti-obesity–related changes observed in obese rats show that repeated oral administration of either quercetin or nanoquercetin resulted in significant reductions in body weight and marked improvements in dyslipidemic parameters. Improvements in lipid homeostasis were reflected by significant decreases in serum total cholesterol, LDL, and triglycerides, together with marked increase in HDL levels in quercetin- or nanoquercetin-administered obese rats, in consistent with previous studies reporting the beneficial role of quercetin in ameliorating lipid metabolism and adipocyte function^44,45^. The reductions in total cholesterol and LDL levels were not statistically significant between quercetin and nanoquercetin, although the decrease was greater in animals receiving nanoquercetin, suggesting a quantitatively enhanced biological response. This lack of significant divergence may reflect overlapping mechanisms by which both forms of quercetin modulate lipid metabolism. Quercetin has been shown to regulate lipid homeostasis through multiple pathways, including anti-inflammatory and antioxidant effects, modulation of lipid transporters such as ABCA1, and activation of peroxisome proliferator-activated receptors (PPARs) and related transcriptional networks involved in fatty acid oxidation and cholesterol efflux, thereby improving lipid handling in metabolic disorders. Meta-analyses in humans and preclinical models report mixed effects of quercetin on plasma lipid parameters, with significant reductions in total and LDL cholesterol in some studies but minimal changes in other indices, indicating that both the dose and duration of treatment influence lipid outcomes^46,47^.

While these findings suggest that nanoquercetin may offer improved efficacy compared with native quercetin, the underlying mechanisms were not directly examined in this study. This is because the pharmacokinetic behavior of quercetin and nanoquercetin has been extensively investigated in prior pharmacological studies, which have demonstrated improved bioavailability and cellular uptake of nanoquercetin relative to quercetin^48–50^. These findings support the growing evidence that nanotechnology-based delivery systems, such as nanoquercetin, can substantially improve the pharmacokinetic properties and biological activity of nutraceuticals. The observed decrease in total protein levels following the 4-week HFD indicates early metabolic disruption and impaired protein homeostasis associated with diet-induced obesity. Both quercetin and nanoquercetin restored total protein levels, with nanoquercetin raising them to values higher than those of the control group. This enhancement in protein level in nanoquercetin obese and non-obese rats may reflect improved hepatic protein synthesis, antioxidant protection, and overall metabolic recovery, consistent with previous reports of quercetin’s hepatoprotective and anabolic effects^51,52^.

Similarly, oral administration of quercetin or nanoquercetin at the tested dose over experimental period significantly modulated adipokine gene expression in HFD-induced obese rats. Specifically, expression of *Adiponectin*, a crucial anti-inflammatory and insulin-sensitizing adipokine, was significantly upregulated, while *Leptin*, commonly elevated in obesity and linked to *Leptin* resistance, was markedly downregulated following quercetin or nanoquercetin administration in obese rats. Notably, nanoquercetin induced a more pronounced modulation in adipokine gene expression than quercetin, resulting in markedly higher *Adiponectin* expression and significantly greater suppression of *Leptin* expression in both hepatic and pancreatic tissues of nanoquercetin-admininstered obese rats compared to quercetin-received obese rats. These findings indicate a superior regulatory effect of nanoquercetin on adipokine signaling pathways. This effect is likely related to the nanoquercetin improved bioavailability and cellular uptake reported in previous pharmacological studies, rather than being directly demonstrated in the present work^48–50^. These results are consistent with prior studies demonstrating quercetin’s ability to restore adipokine balance thereby improving metabolic function and mitigating obesity-related inflammation^53,54^. Overall, the current findings suggest that nanoquercetin may offer a more effective strategy for correcting adipokine dysregulation and enhancing overall metabolic health in obesity.

A critical aspect of this study was the estimation of genomic DNA integrity using the alkaline comet assay, which provided sensitive detection of DNA strand breaks at the single-cell level. In HFD-induced obese rats, a significant increase in DNA damage was observed in both hepatic and pancreatic tissues, as indicated by elevated tail length, %DNA in the tail, and tail moment, hallmarks of oxidative genotoxic stress associated with obesity. Remarkably, daily oral administration of quercetin or nanoquercetin at a dose of 200 mg/kg body weight for four weeks effectively ameliorated these genomic DNA alterations. Both compounds significantly reduced all measured comet assay parameters in obese rats, with values approaching those observed in untreated non-obese control rats, thus restoring genomic stability. Notably, nanoquercetin exhibited a slightly superior genoprotective effect compared to quercetin, likely due to its enhanced bioavailability and tissue penetration. These findings emphasize the capacity of quercetin and particularly nanoquercetin to counteract obesity-induced oxidative DNA damage, a critical pathological feature of metabolic dysfunction^54,55^. This genoprotective effect adds a novel mechanistic dimension to the anti-obesity potential of these flavonoids and highlights the relevance of incorporating DNA integrity as a key molecular endpoint in obesity-related therapeutic investigations.

Histological evaluation further validated the therapeutic and protective roles of both quercetin and its nanoform against obesity-induced tissue damage. In the HFD-fed obese rats, liver and pancreatic sections exhibited severe histopathological alterations indicative of metabolic stress. The liver showed widespread vacuolar degeneration of hepatocytes, hallmarks of hepatic steatosis driven by lipid accumulation and oxidative damage. These structural changes align with previously reported findings on the detrimental effects of HFD on hepatic architecture, including lipid infiltration, hepatocellular ballooning, and inflammatory infiltration^56,57^. Simultaneously, pancreatic tissues from obese rats displayed pronounced islet hyperplasia and necrosis of acinar cells, reflecting impaired pancreatic function and structural integrity. Such features are characteristic of obesity-associated β-cell stress, insulin resistance, and chronic inflammation^58,59^. However, treatment with quercetin at a dose of 200 mg/kg body weight for four weeks partially ameliorated these lesions. In the liver, quercetin administration led to reduced hepatocellular degeneration, while in the pancreas it improved acinar cell morphology and lessened islet hyperplasia. These improvements are consistent with previous studies reporting quercetin’s ability to mitigate hepatic steatosis and preserve pancreatic function via antioxidant and anti-inflammatory mechanisms^60,61^.

Importantly, nanoquercetin demonstrated a markedly enhanced therapeutic effect. HFD obese rats orally administered nanoquercetin at the same dose exhibited near-complete restoration of normal hepatic and pancreatic histoarchitecture, with minimal signs of cellular damage. The improved efficacy of nanoquercetin can be attributed to its enhanced bioavailability, increased solubility, and superior tissue penetration, allowing for more efficient cellular uptake and targeted delivery^62–64^. Its uniform nano-sized particles facilitated deeper tissue access, enabling stronger antioxidant activity and more effective cellular protection and repair.

Moreover, the histological improvements observed with nanoquercetin administration were consistent with functional recovery, as reflected in improved lipid profiles, restored total protein levels, preservation of genomic DNA integrity, and normalization of adipokine gene expression. This comprehensive therapeutic response underscores nanoquercetin’s multi-targeted efficacy, which is crucial in treating multifaceted disorders like obesity that involve simultaneous dysfunction across multiple organs and molecular pathways. Collectively, these findings highlight nanoquercetin’s potential as a next-generation phytotherapeutic agent, capable of overcoming the common limitations of conventional obesity treatments such as poor bioavailability, systemic toxicity, and limited efficacy. In contrast to many synthetic anti-obesity drugs that may cause adverse effects and require long-term usage to maintain results, nanoquercetin offers a safe, sustainable, and organ-protective alternative for managing obesity and its complications.

Despite the exclusive use of male rats represents a limitation of this study, male rats were intentionally selected to minimize variability associated with hormonal fluctuations during the estrous cycle, which are known to influence metabolic and molecular outcomes. Nevertheless, growing evidence indicates that sex-specific differences in metabolism, oxidative stress responses, and drug sensitivity may influence experimental outcomes. Therefore, the findings of this study should be interpreted within this context, and future investigations incorporating both sexes are warranted to comprehensively assess sex-dependent responses and enhance translational applicability.

### Limitations and recommendations/future directions

The results of the current study provide novel insights into the therapeutic potential of quercetin and nanoquercetin against HFD–induced dyslipidemia and genomic DNA damage. Notably, this study demonstrates for the first time the differential therapeutic improvements in both pancreatic and hepatic histopathology, with nanoquercetin showing superior efficacy in preserving tissue structure and reducing cellular injury. However, the study is limited by the absence of functional assessments of β-cell activity and insulin sensitivity, such as fasting blood glucose, serum insulin, HOMA-IR, OGTT, or ITT, which restricts direct correlation between histological improvement and metabolic outcomes. In addition, the experiments were conducted in an animal model, which may not fully reflect human physiology, and only a single dose of each compound was tested, limiting evaluation of dose-dependent effects. Obesity induction was performed during an early-stage model, and longer high-fat diet feeding periods (≥ 8–12 weeks) were not explored to capture chronic obesity progression. Furthermore, detailed histological analysis of the endocrine pancreas, specifically the islets of Langerhans, was not performed in normal control rats. In routine H&E-stained sections, islets in healthy pancreatic tissue were not consistently distinguishable, whereas they were more apparent in disease-affected tissue due to high-fat diet–induced histopathological changes. Consequently, the histological analysis focused primarily on reproducibly identifiable exocrine (acinar) structures. Future studies incorporating specialized staining or immunohistochemistry would be valuable for a more comprehensive assessment of endocrine alterations in metabolic disease models. Future studies should therefore incorporate functional metabolic assays, extended obesity induction protocols, comprehensive adiposity assessments (including longitudinal body weight monitoring and visceral fat quantification), multiple dosing regimens, and validation in additional animal models and clinical settings to strengthen the mechanistic understanding and translational relevance of quercetin and nanoquercetin as potential anti-obesity therapies.

## Conclusion

The present study provides proof-of-concept evidence that both quercetin and its nanoformulation exert beneficial biological effects in a rat model of high-fat diet–induced obesity. Repeated oral administration of either compound at a single dose of 200 mg/kg b.w. for four weeks was associated with significant improvements in several obesity-related parameters, including dyslipidemia, adipokine imbalance, DNA damage indices, and obesity-associated histopathological alterations in hepatic and pancreatic tissues. Across the measured endpoints, nanoquercetin produced quantitatively greater ameliorative effects than native quercetin, suggesting that nanoformulation may enhance the biological activity of quercetin under experimental conditions. However, these differences should be interpreted cautiously, as the study was not designed to assess pharmacokinetics, tissue distribution, or dose–response relationships. Accordingly, mechanistic explanations for the observed quantitative differences remain speculative and require direct experimental validation. In non-obese rats, both formulations were well tolerated, with no evidence of biochemical, genotoxic, or histological adverse effects at the tested dose, supporting their use in short-term experimental settings. While nanoquercetin induced favorable molecular and biochemical changes in healthy animals, these findings should not be interpreted as evidence of preventive or clinical efficacy. The study provides clear evidence of protective effects on exocrine pancreatic architecture under high-fat diet conditions; however, assessment of the islets of Langerhans was limited due to their poor visibility in normal pancreas. Thus, the conclusions primarily reflect exocrine tissue changes, and further studies are needed to evaluate potential endocrine pancreatic effects more comprehensively. Overall, the findings demonstrate that nanoquercetin merits further investigation as a modified formulation of quercetin with enhanced biological activity in preclinical obesity models. Future studies incorporating longer treatment durations, multiple dose levels, pharmacokinetic analyses, and mechanistic validation, followed by appropriately designed translational studies, are necessary to determine its therapeutic relevance and clinical applicability.

## Data Availability

The datasets used and/or analyzed during the current study are available from the corresponding author on reasonable request.
